# FucR Functions as a Transcriptional Regulator for L-Fucose Utilization in *Campylobacter jejuni*

**DOI:** 10.3390/microorganisms13102364

**Published:** 2025-10-14

**Authors:** Wayne T. Muraoka, Nicholas Lizer, Peng Liu, Zhangqi Shen, Qingqing Xia, Muslum Ilgu, Qijing Zhang

**Affiliations:** 1 Department of Veterinary Microbiology and Preventive Medicine, College of Veterinary Medicine, Iowa State University, 1130 Patterson Hall, Ames, IA 50010, USA; 2 Department of Statistics, Iowa State University, Ames, IA 50011, USA; 3 Roy J. Carver Department of Biochemistry, Biophysics and Molecular Biology, Iowa State University, Ames, IA 50011, USA

**Keywords:** *Campylobacter*, fucose utilization, gene regulation, adaptation

## Abstract

*Campylobacter jejuni* is an enteric pathogen and a major cause of foodborne illness worldwide. It has been shown that *C. jejuni* primarily utilizes amino acids as its preferred energy source, but its ability to utilize L-fucose can grant a competitive advantage during intestinal colonization. In *C. jejuni*, fucose utilization is encoded by a variable region named plasticity region 2 (PR2); however, the regulatory mechanism for the region remains unknown and is investigated in this study. Genomic sequence analysis revealed that immediately upstream of the fucose utilization operon is a putative IclR-type transcriptional regulator, *cj0480c* (named *fucR* here). To determine whether *fucR* regulates the expression of the fucose utilization operon, we generated a knock-out mutant of *fucR*. RT-PCR and microarray analysis found that all the genes in the operon were polycistronic and significantly upregulated in the *fucR* mutant compared with their expression in the wild-type strain. In the presence of fucose, expression of the fucose utilization genes was induced in the wild-type strain but no longer inducible in the *fucR* mutant, suggesting that FucR functions as a repressor for the fucose utilization operon. To determine whether FucR directly or indirectly regulates the fucose utilization operon, a 6xHis-tagged full-length FucR was produced in *Escherichia coli*, and the purified recombinant FucR was used in electrophoretic mobility shift assay, which demonstrated that FucR bound specifically to the promoter region of the fucose utilization operon. Together, these results indicate that the L-fucose utilization operon in *C. jejuni* is directly regulated by FucR, which functions as a transcriptional repressor and modulates the expression of the operon in response to fucose.

## 1. Introduction

*Campylobacter jejuni* is among the leading causes of bacterial food-borne illness worldwide, resulting in significant morbidity and economic loss due to health care costs and reduced worker productivity [1]. In addition to the acute symptoms of campylobacteriosis, which include fever, cramping, and diarrhea, severe sequelae such as paralytic disorders Guillain–Barré and Miller Fisher syndromes are also associated with *C. jejuni* infection [2,3]. Human exposure to *C. jejuni* occurs mainly through consumption of contaminated food, primarily poultry, milk or water [4]. As a foodborne pathogen, *Campylobacter* has acquired the ability to survive and thrive in both food-producing animals and the human host [5]. Understanding the pathogen’s adaptive response to nutrient availability in various environments may offer insights into ameliorating the public health burden of *C. jejuni* infection.

*Campylobacter* is unable to catabolize glucose for energy generation due to lacking the 6-phosphofructokinase required for the irreversible phosphorylation of fructose-6-phosphate to fructose-1,6-diphosphate during glycolysis [6,7]. Strain-specific utilization of L-fucose by *C. jejuni* has been reported, highlighting the metabolic diversity of this important pathogen [8,9]. Fucose is a prominent carbohydrate component of eukaryotic glycoproteins, comprising 4–14% of mucin’s total oligosaccharide content [10]. Given that *C. jejuni* is an enteric pathogen, utilization of this substrate provides an advantage for intestinal colonization. Across strains isolated from various host species, approximately 45.5% of *C. jejuni* isolates have the genetic capacity for growth on fucose [9,11]. This phenotype has been linked to a plasticity region of the chromosome (PR2), and gene expression of *fucP* (fucose permease) is induced by fucose in a dose-dependent manner [9]. A recent study conducted Netherlands revealed that PR2 was significantly associated with *C. jejuni* isolates from the human host [11]. Strains containing *fucP* were also linked to livestock isolates and secondarily associated with less severe campylobacteriosis [12]. Conversely, a separate investigation into the interplay between fucose metabolism in *Campylobacter* and the fucosidase activity of *Bacteroides fragilis* indicated that *C. jejuni* was dependent on this enzymatic activity to utilize fucose, leading to an increase in invasiveness with intestinal cells [13]. Another study also linked PR2 with higher invasion and fibronectin binding efficacy of *Campylobacter* [14].

The ability to sense and transcriptionally respond to fucose is likely an adaptive mechanism for nutrient utilization in the environment, such as niches encountered within the host. Indeed, the intestinal mucus is rich in fucosylated glycans, and mucin has a significant impact on *C. jejuni* physiology [13,15]. Genes in the PR2 have been studied and identified as genes required for the utilization of fucose [9]. Recent investigations into this metabolic pathway indicate that *C. jejuni* utilizes a non-phosphorylative pathway for fucose utilization, as opposed to the phosphoralative pathway used in other bacteria such as *E. coli* [16]. This metabolic pathway is hypothesized to rely on the genes cj0482-cj0483, which code for an altronate hydrolase for conversion from L-fuconate to L-2-keto-3-deoxyfuconate [16]. Gene *cj0485*, termed FucX, was shown to function as a fucose dehydrogenase able to reduce both L-fucose and D-arabinose and confer chemotaxis [17,18]. It was also demonstrated that *Campylobacter* prioritized the utilization of amino acids over fucose or arabinose, further highlighting the hierarchy of metabolic regulation [17].

Several regulatory proteins have been characterized that allow *C. jejuni* to sense and respond to external stimuli, including iron, phosphate, aspartate, temperature, and nitrosative, aerobic, osmotic, antibiotic and oxidative stresses [19,20,21,22,23,24,25,26]. Transitory gene expression in different environments may afford the organism an adaptive mechanism to cope with stresses and nutrient availability. Another example of regulated gene expression contributing to the adaptive process of *C. jejuni* is the expression of the CmeABC efflux pump. Bile salts induce this RND-type efflux pump by interfering with the function of the CmeR repressor protein [27], leading to overexpression of *cmeABC* and bile resistance required for the colonization of chicken intestines [28]. In other bacteria, the genes involved in fucose catabolism are inducible and under the control of the transcriptional regulator, termed FucR. In *E. coli*, FucR is a member of the DeoR family of regulators, and functions as an activator for the divergently transcribed *fucPIKU* and *fucAO* operons [29]. Fuculose-1-phosphate, a metabolite of FucK, is the inducer of the FucR regulon [30]. In contrast, FucR in *Bacteroides thetaiotaomicron* functions as a repressor for the *fucRIAK* operon [31]. Direct binding of fucose to FucR in *B. thetaiotaomicron* inhibits DNA binding, allowing for increased gene expression. The *C. jejuni* NCTC 11168 genome does not possess a homolog of the DeoR family regulator, yet *fucP* is induced by fucose in a dose-dependent manner [9]. Instead, an IclR-type transcriptional regulator is predicted to be encoded by the PR2 locus, suggestive of a fucose regulatory mechanism that differs from other bacteria.

IclR-type transcriptional regulators are known to be involved in several metabolic processes and are capable of interacting with transcriptional effector molecules through DNA binding [32]. A prominent feature of this family of regulators is a sequence-specific profile that is not associated with the helix–turn–helix DNA binding motif of the N-terminus [33]. The ligand binding domain is located at the C-terminus, and conformational changes in this region alter DNA binding. Members of the IclR family of regulators can function as both transcriptional activators and repressors within the cell, allowing for coordinated gene expression through a single protein. In this report, we describe the inducible expression and regulation of the fucose regulon in *C. jejuni* NCTC 11168 through an IclR-type transcriptional repressor, named FucR in this study

## 2. Materials and Methods

### 2.1. Bacterial Strains and Growth Conditions

The bacterial strains and plasmids used in this study are listed in Table 1. *Escherichia coli* DH5α was routinely cultured in lysogeny broth or on lysogeny agar at 37 °C. *C. jejuni* strains were cultured on Mueller Hinton (MH) broth or agar at 37 °C microaerobically (85% N_2_, 10% CO_2_, 5% O_2_) in a Model 3130 gas incubator (Forma Scientific, Pittsburgh, PA, USA) or AnaeroPack jar (Thermo Fisher Scientific, Waltham, MA, USA).

### 2.2. Construction and Complementation of Mutants

Isogenic mutants were constructed in the NCTC 11168 background using the inverse PCR strategy described previously [36]. Briefly, *cj0480c* was PCR-amplified from NCTC 11168 using primers cj0480c_L and cj0480c_R, an annealing temperature of 52 °C, and an extension time of 2 min for 35 cycles. The PCR product was cloned into pGEM-T to produce plasmid p*cj0480c* and introduced into chemically competent *E. coli* DH5α. The recombinant plasmid was isolated and used as a template for inverse PCR with primers icj0480c_L and icj0480c_R, deleting an internal fragment of 458 nucleotides of *cj0480c*. The *cat* cassette was PCR-amplified with primers cat_L and cat_R from pUOA18 [35]. The inverse PCR products and *cat* amplicons were purified by ethanol precipitation and digested with BamHI and BglII (New England BioLabs, Ipswich, MA, USA), respectively. The digested products were ligated together and used to transform chemically competent *E. coli* DH5α. The resulting recombinant plasmid (pWM15) was purified and used to transform *C. jejuni* NCTC 11168 to produce the mutant strain CjWM116a (Table 1) as described previously [9]. Complementation of strain CjWM116a was accomplished as previously described [37]. Briefly, the complementing ORF was amplified from *C. jejuni* NCTC 11168 using primers KIcj0480c_L and KIcj0480c_R with annealing at 50 °C and an extension time of 2 min. The ethanol-purified amplicon was digested with AvrII and ligated to pRRK, which was digested with XbaI to produce pRRKWM14. This plasmid transcribes the *cj0480c* ORF in the same transcriptional direction as the ribosomal genes. The transformation of the strain CjWM116a with pRRKWM14 produced the complemented strain CjWM233b (Table 1).

### 2.3. Confirmation of Polycistronic Transcript

**Figure 1:**
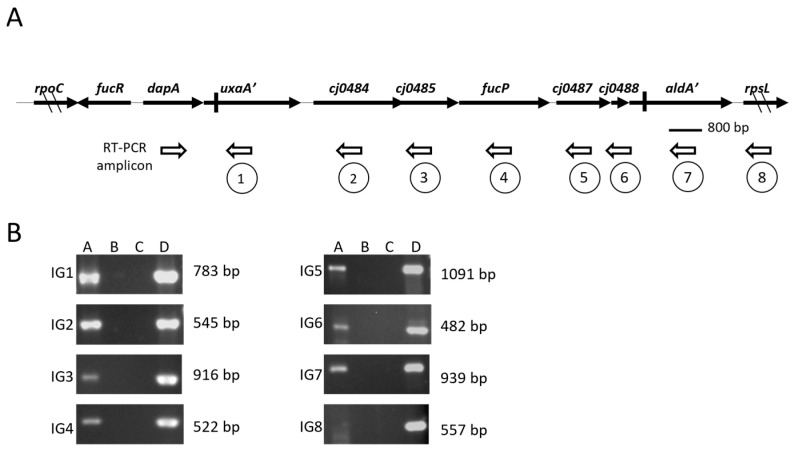
Co-transcription of the genes in the PR2 locus. (**A**) Schematic representation of the PR2 locus in *C. jejuni* NCTC 11168. Solid arrows depict open reading frames. Vertical bars indicate locations of predicted frameshift mutations resulting in premature translational termination. The diagonal hatch marks indicate that the full length of the ORF is not shown. Bottom open arrows indicate primer direction, and numbers 1–8 below each individual reverse primer correspond to RT-PCR amplicons as related to (panel **B**). (**B**) RT-PCR of intergenic regions depicted in (panel **A**). Lane A: RT-PCR with cDNA as template, lane B: no RT controls, lane C: no template controls, lane D: genomic DNA control.

RT-PCR was performed on total RNA extracted from wild-type NCTC 11168 grown at 37 °C, under microaerophilic conditions, for 4h, and in the presence of fucose [9]. cDNA was synthesized by reverse transcription using the primers indicated in Table 2. The intergenic sequences (IG) indicated by the encircled numbers in Figure 1 were PCR-amplified using the corresponding primer pairs in Table 2.

### 2.4. Semi-Quantitative Reverse Transcription PCR

The relative fucP transcript abundance was measured for both the wild-type NCTC 11168 and mutant strains by SYBR green RT-PCR as described previously with the following modifications [9]. L-fucose (Sigma, St. Louis, MO, USA) was added from a 2.5 M frozen stock solution to fresh cultures to a final concentration of 25 mM. All cultures with or without added fucose were incubated at 37 °C under microaerobic conditions. At 0, 1, 2, 3, and 4 h of incubation, 1 mL aliquot was removed from each culture and the total RNA was extracted using the RNeasy Plus Mini kit (Qiagen, Hilden, Germany). The experiment was repeated independently four times. For statistical and graphical purposes, R values (ratio of fucP transcript abundance) were log_2_-transformed. Pair-wise comparisons of fold-differences were evaluated by two-sample *t*-tests. The Holm correction was applied to correct for family-wise error rate associated with multiple testing [38].

### 2.5. Transcriptomic Analysis of Fucose Induction in NCTC 11168 and Cj0480c Mutant

Global transcriptional profiling was performed by microarray analysis according to the manufacturer’s recommendations (JCVI v4.0, J. Craig Venter Institute, La Jolla, CA, USA) with modifications. Briefly, wild-type and Cj0480c mutant strains were grown in MH broth with or without fucose (25 mM final concentration) as described above for qRT-PCR analysis. After 4 hr of incubation, the entire 10 mL culture was harvested by centrifugation and RNA extracted using the RNeasy Plus Mini kit (Qiagen, Hilden, Germany). RNA was DNaseI-treated (DNA-free, Thermo Fisher Scientific, Waltham, MA, USA), and 5 μg of total RNA was labeled with either Cy3 (test condition) or Cy5 (reference condition) for 2 h (Amersham Biosciences, Piscataway, NJ, USA). The wild-type strain grown in the absence of fucose served as the reference condition, whereas the wild-type strain grown with fucose or the Cj0480c mutant strain served as the test conditions. The entire procedure was repeated a total of four times with a dye swap introduced in alternating experiments. Slides were scanned using ScanArray 5000 (Perkin Elmer, Boston, MA, USA), and fluorescence intensity values were acquired using ImaGene 7.0 software (BioDiscovery, El Segundo, CA, USA). Median background corrected fluorescence intensities were LOWESS-normalized and median-centered. Statistical significance was assessed for each probe by moderated *t*-test using the limma 2.0 package (R, The R project for Statistical Computing, Vienna, Austria). The false discovery rate for each probe was controlled for by applying the R package’s q-value [39]. The data discussed in this publication have been deposited in NCBI’s Gene Expression Omnibus [40] and are accessible through GEO Series accession number GSE46808 and GSE46752 (http://www.ncbi.nlm.nih.gov/geo/query/acc.cgi?acc=GSE46808 and http://www.ncbi.nlm.nih.gov/geo/query/acc.cgi?acc=GSE46752) (Last updated 2 January 2014, access date 9 October 2025).

### 2.6. Construction and Production of 6x His-Tagged Cj0480c

N-terminal 6xHis tagged cj0480c was constructed in plasmid pTrcHis (Thermo Fisher Scientific, Waltham, MA, USA). The cj0480c sequence was isolated from *C. jejuni* NCTC 11168 via genomic isolation kit (Promega, Madison, WI, USA) and then amplified by primers cj0480cBamHI and cj0480cEcoRI (Table 2). Both the cj0480c amplicon and pTrcHis plasmid were digested with BamHI and EcoRI. Ligation was carried out with T4 DNA ligase (New England Biolabs, Ipswich, MA, USA) and was subsequently transformed into *E. coli* DH5α competent cells per manufacture instruction (Sigma-Aldrich, Burlington, MA, USA). Transformants were grown in LB broth containing 100 µg/mL ampicillin, and then plasmids were extracted by utilizing the QIAprep Spin Miniprep kit (Qiagen, Hilden, Germany). The purified plasmid was analyzed via standard agarose gel electrophoresis and Sanger sequencing to confirm the cloned *cj0480c* sequence and then transformed into *E. coli* Origami B (DE3) cells (Sigma-Aldrich, Burlington, MA, USA). The Origami B transformants possessing pTrc-cj0480c were grown overnight at 37 °C and then inoculated into 500 mL of fresh Terrific Broth (containing 100 mg/mL ampicillin) at an OD_600_ of 0.01. The culture was incubated at 37 °C with shaking until reaching an OD_600_ of 0.8. IPTG was then added to a final concentration of 0.4 mM, and the induction of Cj0480c expression was allowed to occur overnight at room temperature with shaking. Protein purification was conducted using Ni-NTA (Qiagen, Hilden, Germany) resin according to the manufacturer’s instructions. Briefly, bacterial cells were pelleted and sonicated in lysis buffer containing 1 mM PMSF (Thermo Fisher Scientific, Waltham, MA, USA), 1 mg/mL of lysozyme (Thermo Fisher Scientific, Waltham, MA, USA), and 5 µg/mL of DNase1 (New England BioLabs, Ipswich, MA, USA). The sonicated sample was centrifuged at 16,773× *g* at 4 °C for 15 min. The resulting supernatant was combined with 2 mL of Ni-NTA resin and incubated for 4 h at 4 °C. The incubated resin was loaded onto a chromatography column (Thermo Fisher Scientific, Waltham, MA, USA), which was first washed with increasing amounts of imidazole (10 mM, 20 mM and 40 mM) in washing buffer and then eluted with the elution buffer containing 300 mM imidazole. Protein concentrations were determined using NanoDrop (Thermo Fisher Scientific, Waltham, MA, USA), and the purity of the protein preparation was analyzed by SDS-PAGE. Finally, Western blotting was performed by utilizing anti-6 x His monoclonal antibodies (Invitrogen, Waltham, MA, USA) and the SeraCare KPL 4 CN Peroxidase Substrate System (Milford, MA, USA). Gel images were captured by using a ChemiDoc (Bio-Rad, Hercules, CA, USA).

### 2.7. Electrophoretic Mobility Shift Assay (EMSA)

EMSA was performed as previously described with the following specifications [41]. Purified Cj0480c (named FucR here) was desalinated and exchanged into a binding buffer utilizing Bio-Spin p-6 gel columns (Bio-Rad, Hercules, CA, USA). The binding buffer consisted of 50 mM Tris-HCl, 150 mM KCl, and 10 mM MgCl_2_ at pH of 7.4. Final protein concentration was determined via Qubit (Thermo Fisher Scientific, Waltham, MA, USA). DNA fragments were generated by PCR utilizing the genomic DNA of *C. jejuni* NCTC 11168 as template and two separate primer pairs (Table 2): fucR_promoter_L and R to amplify the promoter region of *fucR* and dapA_internal_L and R to amplify an internal section of *cj0481*. In the reactions, 30 ng of each individual DNA fragment and increasing amounts of FucR, at intervals of 1.4 µg from 0 µg to 11.2 µg, were mixed and allowed to incubate at room temperature for one hour. EMSAs were carried out on 4–20% gradient PAGE gels (Bio-Rad, Hercules, CA, USA) and run for 90 min at a constant 90 V on a Bio-Rad PowerPack. Gels were stained with ethidium bromide (Sigma, St. Louis, MO, USA) for 15 min and then imaged on a ChemiDoc (Bio-Rad, Hercules, CA, USA). To determine whether L-fucose affected FucR binding, increasing concentrations of L-fucose from 0.1 mM to 100 mM were added to reactions consisting of 2 µg of FucR and 30 ng of DNA. Reactions were subsequently analyzed by EMSA.

## 3. Results

### 3.1. Genetic Organization and Co-Transcription of PR2

The genes associated with fucose utilization in *C. jejuni* have been linked to a cluster of ORFs located in a plasticity region of the chromosome (PR2) [9]. This 11 ORF cluster encodes the fucose permease (*fucP*), but homologous genes known to be involved in fucose metabolism in other bacteria are notably absent. *cj0480c* encodes a predicted transcriptional regulator and is transcribed divergently from the rest of the locus with a 229-base intergenic region (Figure 1A). ORFs *cj0481-cj0490* are tightly clustered with predicted overlapping translational stop and start codons. Intergenic spaces, when present, ranged from 9 to 60 bases in length, suggesting ORFs *cj0481-cj0490* are transcribed as a polycistronic message. RT-PCR amplification of the intergenic or overlapping regions supports this assumption (Figure 1B). Additionally, as discussed below, coordinated fucose-induced gene expression further corroborates co-transcription of *cj0481*-*cj0490* as a polycistronic message.

### 3.2. PR2 Harbors a Transcriptional Regulator That Senses and Responds to Fucose

**Figure 2:**
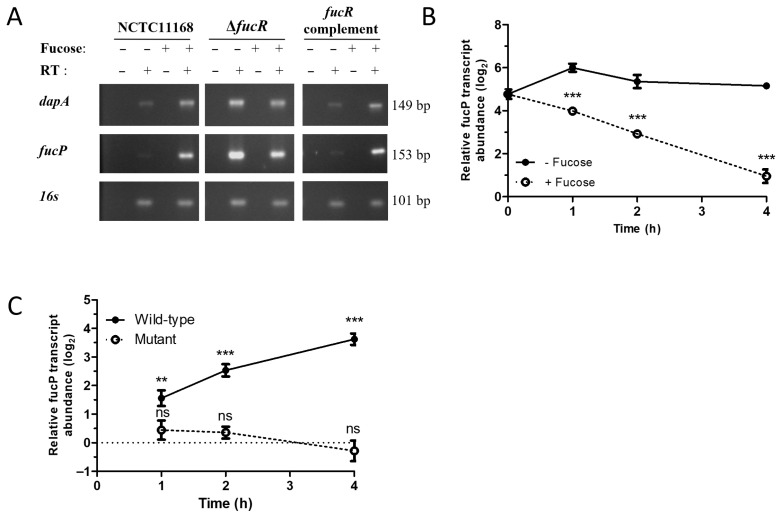
FucR (Cj0480c) regulates the expression of genes associated with fucose utilization. (**A**) RT-PCR for *dapA*, *fucP* and 16s RNA transcripts from wild-type NCTC 11168, the *fucR* mutant (CjWM116a), and complemented (CjWM233b) strains. Fucose +: cultures were supplemented with 25 mM fucose; fucose-: cultures were not supplemented with fucose; RT +: reverse transcribed with SuperScript; RT-: no SuperScript reverse transcriptase control. (**B**,**C**) Semi-quantitative RT-PCR measuring relative *fucP* expression in the *fucR* mutant and wild-type strains grown with or without fucose. In (panel **B**), each data point represents the mean log_2_ fold-difference of *fucP* transcript abundance (mutant vs. wild type) ± SEM. Cultures with and without fucose are represented by dashed and solid lines, respectively. Statistical differences between cultures grown with and without fucose are indicated by asterisks. In (panel **C**), each data point represents the mean log_2_ fold-difference of *fucP* transcript abundance (with fucose vs. without fucose) ± SEM. Significant *fucP* induction by fucose was determined by a one-sample *t* test with a hypothetical mean of 0. ns: *p* > 0.05, **: *p* < 0.01, ***: *p* < 0.001.

Fucose has been shown to significantly up-regulate *fucP* in a dose-dependent manner [9], suggestive of a transcriptional regulatory mechanism that senses and responds to this growth substrate. Annotation of the NCTC 11168 genome identified *cj0480c* as a putative IclR-type transcriptional regulator [34,42], which is named *fucR* in this study (Figure 1A). A *fucR* mutant was constructed in the NCTC 11168 background by disrupting the gene with a chloramphenicol resistance cassette. In contrast to the fucose-inducible gene expression of the wild-type strain, this mutant demonstrated constitutive, high levels of *fucP* and *dapA* expression (Figure 2A). Complementation of the mutant restored inducible gene expression to wild-type levels. A quantitative assessment of gene expression was performed using a SYBR green-based real-time RT-PCR assay, where R represents the log_2_ *fucP* transcript abundance of the mutant stain compared to the wild-type (Figure 2B).

**Figure 3:**
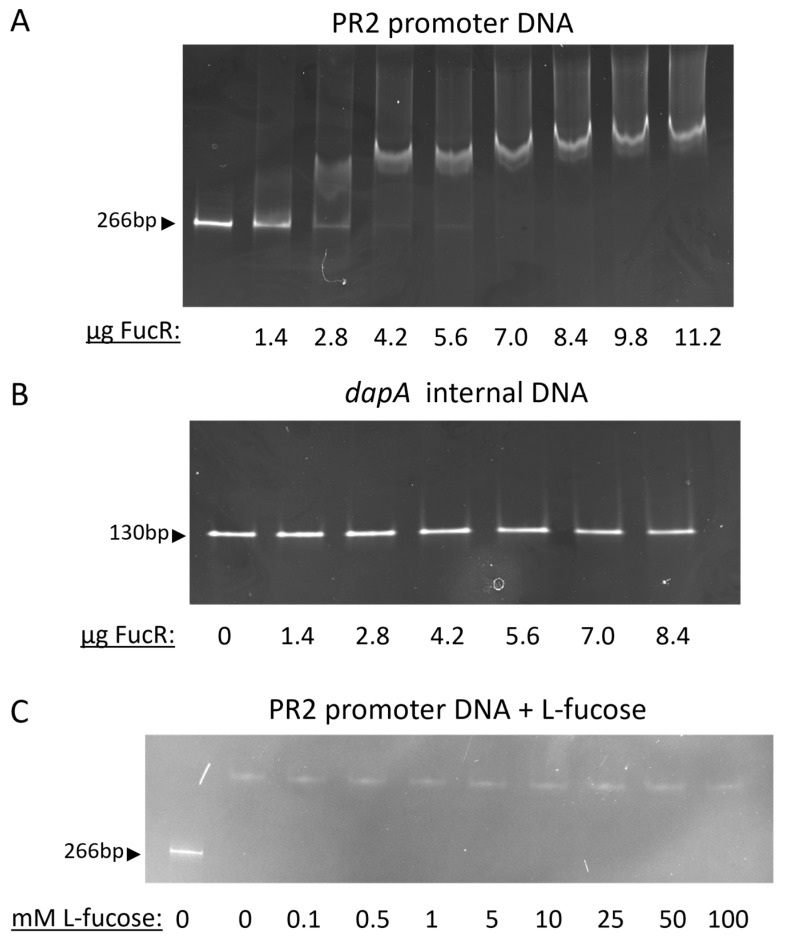
Direct binding of FucR to the promoter DNA upstream of *dapA* (*cj0481*) as demonstrated by EMSA. (**A**) EMSA with the PR2 promoter DNA as the target DNA. Each lane contains 30 ng of DNA and increased amounts of purified FucR, starting at 0 µg in lane one and 1.4 µg in lane two, increasing by 1.4 µg per lane, with the last lane containing 11.2 µg of FucR. The increase in protein concentration correlates with a shift upward from left to right. (**B**) EMSA with an internal section of *dapA* (*cj0481*) as the target DNA. Each lane contains 30 ng of DNA and with increased amounts of purified FucR starting at 0 µg in lane one and 1.4 µg in lane two, increasing by 1.4 µg per lane, with the last lane containing 8.4 µg of FucR in the last lane. The increase in protein concentration demonstrates no shift upward, acting as a negative control. (**C**) EMSA in the presence of increased amounts of L-fucose. The first lane contains only 30 ng of PR2 promoter DNA and each subsequent lane contains 30 ng of PR2 promoter DNA and 2 µg of FucR. Increasing amounts of L-fucose are added to the reactions as denoted below each lane. Each experiment was repeated at least three times and representative gel images are shown.

We observed that at the beginning of the experiment (time 0; Figure 2B), the mutant strain had significantly more *fucP* transcript than the wild-type (R = 4.77 ± 0.21; *p* = 0.0064). This relative abundance of *fucP* transcript was maintained throughout the four-hour experiment in cultures that lacked fucose. In contrast, cultures containing fucose resulted in declining R values in a log-linear, time-dependent manner (Figure 2B). This decrease in R values was due to increased transcription of *fucP* in the wild-type strain in the presence of fucose (Figure 2C). In contrast, transcription of *fucP* in the mutant was not significantly affected by the presence of fucose at any of the time points measured (Figure 2C). Together, the results indicated that FucR functions as a repressor of the PR2 genes and fucose abolishes this repression. To further elucidate the mechanism by which FucR represses the fucose utilization genes, EMSA was conducted to demonstrate the direct interaction between FucR and the putative promoter region situated immediately upstream of *cj0481*(*dpaA*). As demonstrated in Figure 3A, dose-dependent binding of FucR to the promoter DNA resulted in a size shift of the DNA-protein complex band on a polyacrylamide gel under non-denaturing conditions. This shift did not occur with an internal fragment of the *dapA* (cj0481) gene (Figure 3B), which was used as a negative control. This result demonstrates that FucR binds specifically to the promoter DNA. To examine whether fucose inhibits FucR binding, fucose was added to the EMSA reactions. However, no corresponding loss of shifted band occurred (Figure 3C), indicating that presence of fucose in the in vitro EMSA assay did not affect FucR binding to the target DNA.

### 3.3. Transcriptome Analysis of the FucR Mutant and Wild-Type Strain Grown with Fucose

Although the results above demonstrate that FucR regulates genes in the PR2 locus, it is unknown if it also modulates the expression of genes outside PR2. Thus, a competitive microarray analysis was conducted to determine the extent of transcriptional regulation by this protein. A comprehensive transcriptome analysis may also provide valuable insights into the mechanism of fucose utilization by analyzing genes that are differentially expressed during growth in the presence of fucose. cDNA from the wild-type strain grown without fucose (reference condition) was co-hybridized with cDNA from either the fucose-grown wild-type strain or the *fucR* mutant (test conditions).

Comparative hybridization between wild-type and the *fucR* mutant revealed a restricted number of differentially expressed genes (q-value ≤ 0.05). In all, 16 ORFs were differentially expressed: 2 ORFs were downregulated while 14 ORFs were upregulated (Table 3) in the *fucR* mutant. Of the 14 upregulated ORFs, 10 belonged to the PR2 locus. As expected, all probes associated with the structural genes of the PR2 locus were upregulated with large fold-change values, indicative of a common regulatory mechanism and consistent with a regulon structure. Non-PR2 ORFs identified as differentially expressed showed smaller fold-change values (log_2_ fold-change range −1.07 to 1.55).

Transcriptome analysis of the wild-type strain grown with fucose identified 31 ORFs as differentially expressed (q-value ≤ 0.05); 13 ORFs, in addition to all 10 PR2 structural genes, were upregulated, while 8 ORFs were downregulated (Table 3). Interestingly, although *fucR* is within the PR2 locus, it was not identified as differentially expressed by microarray (q-value = 0.70, log_2_ fold-change = −0.03) or qRT-PCR (log_2_ fold-change = 0.31), suggesting that transcription of the regulator is not responsive to growth with fucose. Similar to the *fucR* mutant transcriptome analysis, fold-change values were smaller for non-PR2 ORFs. Non-PR2 ORFs that were significantly upregulated were associated with protein synthesis and aspartate/glutamate and succinate metabolism, suggestive of carbon assimilation of fucose through the TCA or an amino acid pathway. *cstA* and *cj1548c* were confirmed by RT-PCR to be significantly down-regulated at −0.473 and −0.523, respectively. The *cstA* homolog in *E. coli* encodes a peptide transporter and is induced by carbon starvation through cAMP levels.

Three ORFs (*porA*, *aspB* and *cj1064*) were up-regulated under both the fucose-grown wild-type and the *fucR* mutant conditions, suggestive of a common regulatory and/or metabolic mechanism. *porA* encodes the major outer membrane protein (MOMP), a porin that facilitates the transfer of hydrophilic solutes into the cell [43]. *aspB* encodes an aspartate aminotransferase, the enzyme that catalyzes the transamination of glutamate and aspartate with its corresponding α-keto acid [44]. *cj1064* encodes a possible NAD(P)H nitroreductase; however, the gene appears to have a single base deletion resulting in a frame shift mutation [34]. Investigations into *cj1064* have shown that it maintains transcription, which could hint at its continued functionality and was upregulated in response to oxidative stress [45,46].

## 4. Discussion

The metabolic diversity among isolates of *C. jejuni* is evidenced by strain-specific growth on glutathione, glutamine, asparagine and fucose [8,9,47]. These strain-specific metabolic capabilities may allow for adaptation to niche specialization in particular environments. Isogenic mutants that are deficient in fucose utilization are at a competitive disadvantage to the wild-type during intestinal colonization in fucose-rich environments. Recent epidemiological studies examined the distribution, host association and relatedness of strains with different metabolic traits. The frequencies of strains that have the genetic capacity to metabolize fucose (*fucP*^+^) range from 30 to 65% depending on the study and source of isolates [48,49]. These strains appear to be phylogenetically related and demonstrate strong association with livestock species [9,12] and the MLST-21 clonal complex [49].

Fucose utilization by *C. jejuni* appears to be evolutionarily distinct from other bacteria, as this organism lacks the early genes known to be required for fucose dissimilation. Specifically, homologs necessary for growth on fucose, including the fucose isomerase, kinase, aldolase and 1,2-propanediol oxidoreductase, are not predicted to be encoded by the *C. jejuni* NCTC 11168 genome. Instead, *C. jejuni* growth on fucose requires the PR2 locus with *fucP*-*cj0488* necessary for the phenotype [9]. In both pathogenic and commensal bacteria, genes associated with fucose utilization are inducible and under the control of a member of the DeoR family of transcriptional regulators [30,31]. The induction of the *fuc* regulon in other bacteria occurs either through activation or repression. For example, in *E. coli*, the FucR-fuculose-1-phosphate complex activates the *fucPIKU* and *fucAO* operons [29]. In contrast, FucR in *B. thetaiotaomicron* functions as a repressor for the *fucRIAK* regulon, and binding of fucose to FucR inhibits transcriptional repression [31]. Although no DeoR homolog of FucR is found in the *C. jejuni* NCTC 11168 genome, *fucP* is induced by fucose. In this study, we demonstrate that *fucR* (*cj0480c*), which is transcribed divergently from the structural genes in the PR2 locus (Figure 1A), functions as a repressor for the fucose-utilization genes. Interestingly, although *fucR* regulates the structural genes necessary for growth on fucose, it shares little amino acid sequence similarity to fucose regulators of other bacteria. Rather, the FucR of *C. jejuni* NCTC 11168 clusters closely with the IclR family of transcriptional regulators. Residues 7 to 89 of this protein are predicted to form a helix–turn–helix DNA binding motif, suggesting *fucR* regulates the expression of PR2 structural genes through DNA binding. Indeed, this was demonstrated via EMSA, highlighting the specific and direct interaction of FucR with the promoter DNA (Figure 3). However, the addition of L-fucose to the binding reaction did not result in the release of FucR from the promoter (Figure 3C). The result could be explained in several ways. The EMSA was conducted in a simple mixture of DNA and FucR in vitro, which may not represent the actual environment within bacterial cells, where binding of fucose to FucR might need another helper factor. Alternatively, fucose might not be the actual ligand of FucR. Instead, an intermediate metabolite of fucose could serve as the ligand that binds to FucR and hence releases its inhibition on the expression of the fucose utilization genes.

Under uninduced conditions, *cj0481-cj0490* gene expression is significantly higher in the *fucR* mutant strain than in the wild-type strain, consistent with the notion that FucR functions as a transcriptional repressor in *C. jejuni*. This result corresponds with data indicating that the deletion of *fucR* leads to constitutive overexpression of the genes in the PR2 locus (Figure 2 and Table 3) and an increase in fucose uptake [17]. The FucR regulon is almost exclusively restricted to the PR2 locus of the NCTC 11168 genome as identified by microarray transcriptional profiling. Six non-PR2 ORFs were deemed differentially expressed by microarray; however, they could not be independently confirmed by qRT-PCR (Table 3), suggestive of false discovery in the microarray. Consistent with its proximity to the plasticity region, FucR appears to be a specific regulator for the PR2 locus and does not appear to play a role in regulating genes outside of this locus.

Previous investigation of *C. jejuni* gene expression identified more genes as differentially expressed compared to our current study [8]. This apparent discrepancy may be attributed to different experimental designs. The previous design addressed the question of gene expression when grown on different growth substrates, while our current design addressed gene expression under identical growth conditions with fucose supplementation as the only variable. Whereas the former design would identify differential gene expression due to growth on either pyruvate or fucose, the latter design could only identify genes that are differentially expressed due solely to fucose. Nevertheless, common themes emerged from both studies that may provide insights into the mechanism of fucose utilization in *C. jejuni*. Firstly, all structural PR2 genes were found to be significantly upregulated under the fucose-grown condition. Upregulation of PR2 was also shown by proteomic analysis [14], indicating the congruence between the gene transcriptional data and the protein production data. Secondly, *aspA*, *aspB*, *sdhA* and *sdhB* were all significantly upregulated, suggesting aspartate/glutamate and succinate metabolic pathways contribute to *C. jejuni* growth on fucose. A previous study investigating the metabolic effect of fucose on *Campylobacter* growth using HPLC analysis also demonstrated increased succinate production along with pyruvate [50]. AspB is involved in the transamination of oxaloacetate and glutamate to aspartate and 2-ketoglutarate. However, the role of aspartate/glutamate transamination in the fucose phenotype is unknown and a second transaminase homolog is also encoded in the *C. jejuni* genome (*cj0150c*) [44].

The gene *porA* was identified as significantly upregulated under both fucose-grown and *fucR* mutant conditions. PorA is the major outer membrane protein (MOMP) in *C. jejuni* and facilitates the import of hydrophilic solutes into the cell [43]. This porin is differentially regulated under various growth conditions, including temperature and pH [51]. Fucose potentially enters the cell through this porin; however, the essential function of this protein [52] hinders the creation of specific isogenic mutants needed to test this hypothesis. An alternative strategy to mutagenesis may be the gene-silencing approach used previously [53]. *cstA* transcriptional expression was confirmed to be downregulated by qRT-PCR (Table 3). In *E. coli*, CstA likely functions as a peptide transporter during carbon starvation and is transcriptionally activated by cAMP levels through catabolite repression [54,55]. *C. jejuni* does not possess an adenylyl cyclase homolog (*cya*) and therefore likely does not exhibit a form of catabolite repression that resembles *E. coli*. Despite this, *C. jejuni* may still prioritize utilization of growth substrates. Work by Garber et al. indicated L-serine, L-glutamic acid, and L-aspartic acid reduced L-fucose uptake, while *fucP* transcription levels were unchanged by the addition of L-serine to the growth medium [17]. This, combined with FucR’s DNA binding being insensitive to the addition of L-fucose, hints at a possibly more complex regulatory system than direct catabolite repression.

In summary, *C. jejuni* possesses a fucose utilization system under the repression of FucR. This regulation affects the expression of every gene within the PR2 locus and is responsive to the presence of fucose. FucR directly represses the fucose utilization system by binding to the promoter sequence immediately preceding the PR2 operon, and deletion of *fucR* allowed for constitutive expression of the PR2 genes. Although the expression of the PR2 genes is responsive to fucose, FucR’s DNA binding was not inhibited by fucose when tested via EMSA, suggesting that fucose might not directly interact with FucR. How fucose releases the repression of FucR remains to be determined in future studies.

## Figures and Tables

**Table 1:** Bacterial strains and plasmids used in this study.

Bacterial Strain or Plasmid	Description or Relevant Genotype	Source or Reference
*E. coli* strain		
DH5α Origami B(DE3)	Plasmid propagation strain Protein expression strain	Invitrogen Sigma-Aldrich
*C. jejuni* strains		
NCTC 11168	Wild-type *C. jejuni*	[34]
CjWM116a	NCTC 11168 derivative, Δ*cj0480c*::*cat*; Cm^r^	This study
CjWM233b	CjWM116a derivative, Δ*cj0480c*::*cat*, *ITS1*::*cj0480c*; Cm^r^, Kan^r^	This study
Plasmids		
pGEM-T	Cloning vector	Promega
pUOA18 pTrcHis	*E. coli-C. jejuni* shuttle vector *E. coli* expression vector	[35] ThermoFisher
p*cj0480c*	pGEM-T::*cj0480c*	This study
pWM15	Δ*fucP*::*cat*; Cm^r^ suicide vector to construct CjWM114a	This study
pRRK	pRR::*aphA3*	[9]
pRRKWM14 pTrc-cj0480c	pRRK::*cj0480c* pTrc::*cj0480c*-6xHis	This study This study

Cm^r^: chloramphenicol resistant, Kan^r^: kanamycin resistant.

**Table 2:** Primer sequences used in this study *.

Primer Name	Primer Sequence (5′ → 3′)
Primers to construct mutant and complement strains	
cj0480c_L	CGCTCAAAGCTTGAGAATCC
cj0480c_R	GTTTATCGCGGACAAGGTGT
icj0480c_L	CGCGGATCCCCAAAGAGTTCCAGCAGGAA
icj0480c_R	CGCGGATCCTCCTCAAATTGAATGTATGGCT
KIcj0480c_L	CGCCCTAGGAAGCTGTTAACTTGGTAAAAATTCG
KIcj0480c_R	CGCCCTAGGAGCCCTTCGGGGCTATATTA
cj0480cBamHI	GGATCGATGGGGATCCATGCATCAGCCCAC
cj0480cEcoRI	GCCAAGCTTCGAATTCTTAATACAGTGTATCTAAATC
fucRpro_L	GCTGATGCATATTGTCTTTATCC
fucRpro_R	GTAAGTAAAGCCGGTAAAGTTCC
dapAintl_L	ATGAAAAAGAATTTATTCGC
dapAintl_R	ACGCTCAAAGCTTG
Primers to amplify intergenic sequences (IG)	
IG1_L	GCATAGGTGGAGTTTTTCCAG
IG1_R	*AAAAACATTGGCATTGCTCC*
IG2_L	TAGCCCAGGAAATATGGCAG
IG2_R	*AAAGGCAAAATACGCCAAGA*
IG3_L	TCGCCTTGCCAATATTTACC
IG3_R	CTTAGCAAAAGCACAAGCCC
IG4_L	TGACCAAAGAATGTTTGCCTT
IG4_R	*AAGTCCATAGCTTACTCCCCA*
IG5_L	GGCTTTTAGCGCAGTTTTTG
IG5_R	TGCGAAAAACCATCAGGAAT
IG6_L	CAGACATGGCTAAAATGGCA
IG6_R	TTCGGCCGTATAATCCATATAA
IG7_L	GACGATGAAAAATTAGAGCA
IG7_R	*TTTCCACCATTTTTGTGTGC*
IG8_L	CAAGGTTTTCATGCAGGCTT
IG8_R	*ACCCTAGTGCAAACTCCCCT*
Primers for RT-PCR	
qfucP_L	GGCTTTTAGCGCAGTTTTTG
qfucP_RT	CAATGCGCCCTAGCATAAAT
cjr01_L	TCCCAGTTCGGATTGTTCTC
cjr01_RT	GTACAAGACCCGGGAACGTA
pdapA_R	GCAAAGCAAAACCCATAGGA
tdapA_L	GCATAGGTGGAGTTTTTCGAG
Primers to confirm microarray	
cj0917c_F	GCGTTTTATCCGTCCAGGTA
cj0917c_R	TTGCCAAAGTTGGAGCTTCT
cj0927_F	GCAGGAACAGAAAGCAGAGG
cj0927_R	TCAAAAGGGAGTTTTCCAGG
cj1548c_F	AGAAGGCTCAAGCGTAGCAG
cj1548c_R	ACACCCATAGCCAAAGCATC
cj0762c_F	AAGCCCTAATTTCAGCCGTT
cj0762c_R	GTTTTTCAAAGGCTTGACGC
cj0087_F	TGGGGAATTGGAAATCTCTG
cj0087_R	CAAAGCCCTAACAAAGCGAG
cj0437_F	AATTGGATCAGGTGGAGCAG
cj0437_R	CCACCCTCTGCCATACAAGT
cj1604_F	CAAGATGCAAAAACTTGCGA
cj1604_R	CCTTCATCCAAAGACGCTGT
cj0169_F	TTCAAATGGGGGCGTATTTA
cj0169_R	TACTTTGACATGAACCGCCA
cj0933c_F	CAAAACACGGTACCACAACG
cj0933c_R	CAATTTGGCGGTAGATCCAT
cj1259_F	GCAGAGCAAGGTGCAGATTT
cj1259_R	AGCAGCAGCACCGTAAAGAT
cj1339c_F	GCAGGCTCAGGTTTTTCAAG
cj1339c_R	CGGCTGCAAAGTCTACATCA

* Added restriction endonuclease sites are underlined. Primer sequences indicated by italics were used in reverse transcription reactions to synthesize cDNA.

**Table 3:** Global transcriptional profiling of the wild-type grown without fucose (reference condition) compared to the wild-type grown with fucose or the *fucR* (*cj0480c*) mutant (test condition).

		Wild-Type + Fucose		*fucR* Mutant	
Gene ^¶^	Function	Microarray log_2_ Fold-Change (*q*-Value) *	qRT-PCR log_2_ Fold-Change (*p*-Value) *	Microarray log_2_ Fold-Change (*q*-Value) *	qRT-PCR log_2_ Fold-Change (*p*-Value) *
*dapA*	putative dihydrodipicolinate synthase	3.799 (1.75 × 10^−10^)		3.781 (1.28 × 10^−7^)	
*cj0488*	conserved hypothetical protein Cj0488	3.673 (4.58 × 10^−8^)		3.600 (1.28 × 10^−7^)	
*uxaA’*	putative altronate hydrolase C-terminus	3.644 (4.09 × 10^−10^)		3.892 (2.06 × 10^−6^)	
*cj0487*	putative amidohydrolase	3.638 (1.28 × 10^−8^)		3.846 (1.83 × 10^−7^)	
*ald’*	putative aldehyde dehydrogenase N-terminus	3.629 (1.75 × 10^−10^)		3.664 (8.21 × 10^−7^)	
*uxaA’*	putative altronate hydrolase N-terminus	3.573 (3.11 × 10^−8^)		3.184 (2.06 × 10^−6^)	
*cj0486*	putative sugar transporter	3.387 (5.18 × 10^−8^)		3.606 (2.06 × 10^−6^)	
*cj0485*	short chain dehydrogenase	3.339 (1.75 × 10^−10^)		3.254 (4.63 × 10^−6^)	
*ald’*	putative aldehyde dehydrogenase C-terminus	2.581 (7.66 × 10^−6^)		2.593 (1.83 × 10^−7^)	
***cj0484***	**putative MFS (Major Facilitator Superfamily) transport protein**	1.847 (4.48 × 10^−4^)	3.961 (0.001)	2.039 (5.23 × 10^−7^)	5.388 (0.000)
*cj1064*	pseudo	1.408 (3.36 × 10^−5^)		1.551 (2.34 × 10^−3^)	
*aspB*	aspartate aminotransferase	1.347 (1.05 × 10^−5^)	−0.089 (0.620)	1.271 (3.59 × 10^−2^)	−0.046 (0.822)
*porA*	major outer membrane protein	0.769 (2.39 × 10^−4^)	−0.052 (0.553)	0.784 (9.61 × 10^−3^)	−0.069 (0.473)
*rrc*	non-haem iron protein	0.701 (3.21 × 10^−3^)			
*aspA*	aspartate ammonia-lyase	0.637 (3.73 × 10^−3^)	0.298 (0.324)		0.067 (0.649)
*rplF*	50S ribosomal protein L6	0.611 (1.22 × 10^−2^)			
*sdhA*	succinate dehydrogenase flavoprotein subunit	0.587 (3.21 × 10^−3^)	0.466 (0.233)		0.636 (0.077)
*rplE*	50S ribosomal protein L5	0.552 (3.11 × 10^−2^)			
*rplX*	50S ribosomal protein L24	0.535 (2.90 × 10^−2^)			
*cj1534c*	putative bacterioferritin	0.533 (1.66 × 10^−2^)			
*sdhB*	putative succinate dehydrogenase iron-sulfur protein	0.524 (2.90 × 10^−2^)			
*ndk*	nucleoside diphosphate kinase	0.486 (2.92 × 10^−2^)			
*rpsF*	30S ribosomal protein S6	0.476 (2.78 × 10^−2^)			
*cj0416*	hypothetical protein	−0.427 (4.01 × 10^−2^)			
*putA*	putative proline dehydrogenase	−0.463 (3.05 × 10^−2^)			
*prsA*	ribose-phosphate pyrophosphokinase	−0.472 (3.40 × 10^−2^)			
***cstA***	**putative integral membrane protein (CstA homolog)**	−0.473 (3.29 × 10^−2^)	−0.199 (0.003)		−0.040 (0.391)
*cj1111c*	putative MarC family integral membrane protein	−0.520 (4.77 × 10^−2^)			
***cj1548c***	**putative NADP** **-dependent alcohol dehydrogenase**	−0.523 (2.78 × 10^−2^)	−0.319 (0.034)		
*aptA*	adenine phosphoribosyltransferase	−0.821 (8.81 × 10^−3^)	−0.028 (0.837)		−0.423 (0.08)
*glnA*	glutamine synthetase	−0.956 (3.33 × 10^−2^)			
*cj0037c*	putative cytochrome C			−0.532 (8.82 × 10^−3^)	
*rpmF*	50S ribosomal protein L32			0.495 (1.28 × 10^−2^)	
*flgH*	flagellar basal body L-ring protein			−1.07 (2.46 × 10^−2^)	

* Results are presented as log_2_ fold-change; thus, positive and negative fold-change values indicate upregulation and downregulation of the gene, respectively. ^¶^ PR2 genes are indicated by underlined text, and bold text indicates that differential expression was confirmed by qRT-PCR.

## Data Availability

Access to microarray datasets: http://www.ncbi.nlm.nih.gov/geo/query/acc.cgi?acc=GSE46808 (accessed on 9 October 2025) and http://www.ncbi.nlm.nih.gov/geo/query/acc.cgi?acc=GSE46752 (accessed on 9 October 2025).
